# The effects of work on the health of nurses who work in clinical surgery departments at university hospitals**^1^**

**DOI:** 10.1590/1518-8345.0763.2743

**Published:** 2016-08-08

**Authors:** Rosângela Marion da Silva, Regina Célia Gollner Zeitoune, Carmem Lúcia Colomé Beck, Milva Maria Figueiredo de Martino, Francine Cassol Prestes

**Affiliations:** 2 PhD, Adjunct Professor, Departamento de Enfermagem, Universidade Federal de Santa Maria, Santa Maria, RS, Brazil.; 3 PhD, Full Professor, Escola de Enfermagem Anna Nery, Universidade Federal do Rio De Janeiro, Rio De Janeiro, RJ, Brazil.; 4 PhD, Associate Professor, Departamento de Enfermagem, Universidade Federal de Santa Maria, Santa Maria, RS, Brazil.; 5 PhD, Volunteer Associate Professor, Faculdade de Ciências Médicas, Universidade Federal de Campinas, Campinas, SP, Brazil.; 6 Doctoral Student, Universidade Federal de Santa Maria, Santa Maria, RS, Brazil. RN, Departamento de Enfermagem, Universidade Federal de Santa Maria, Santa Maria, RS, Brazil.

**Keywords:** Nurses, Male, Occupational Health, Nursing Service, Hospital, Occupational Health Nursing

## Abstract

**Objective:**

to analyze the effects of work on the health of nurses who work in clinical surgery departments at university hospitals in relation to physical, social and psychological suffering and pain.

**Methods:**

a quantitative transversal study was carried out between 2012 and 2013 in four institutions in a state located in the south of Brazil. We studied 65 nurses who responded to questions on their habits. We also obtained sociodemographical information on them as well as conducting an evaluation on work relational damage using an evaluation scale. Associations were checked through the use of the Chi-Sqaure and Fisher's exact test. Correlations were checked using the Spearmann test.

**Results:**

we found that physical ailments persisted and that there were connections between social and psychological pain/suffering and variable physical activities as well as connections with accidents in the work place and the option to work shifts. We noted correlations between social and psychological pain/suffering.

**Conclusion:**

nurses had their health compromised due to their work in clinical surgery departments.

## Introduction

In the context of work in the health service, nurses stand out as professionals who manage other health care workers in the provision of care for patients that need intensive care^1^. Nurses are exposed to certain situations in hospitals which as a result, can impair their health. This is due to how their work is organized: the need to carry out shift work which includes working nights. The authors suggested that this presented risk factors that could affect someone's mental health^2^. Other effects include: becoming overweight or obese ^3^ ^-^ ^4^ ^)^ and difficulty in doing physical activities^3^ which can negatively impact upon social and family relations as well the actual worker. As a result, work place modifications are needed.

Poor physical, material and humans resources are aggravating factors that have contributed in causing physical and mental problems for those working in university hospitals. Health workers and particularly nurses have been gradually becoming ill according to some studies^5^ ^-^ ^7^.

Clinical surgery departments are unique in the sense that they have experienced staff that provide assistance to others and a large amount are teaching staff. Nurses are required to have scientific knowledge and work with dynamism in order to assist people ahead of and after operations in a way that ensures quality of service and that care is given without risking people lives. Nurses often have poor body postures due to the nature of the work and the strength that is required. The above aggravate the situation and in turn there are adverse repercussions for them in relation to their health.

Nurses have been suffering from the impact of poor social and political policies which has not only contributed to poor working conditions but also has resulted in detrimental impacts on their health. They suffer from physical, social and psychological suffering and pain because the official statistics^5^do not reveal their plight.

Physical problems include body pains and biological disorders. Psychological suffering and pain, such as negative thoughts about one's self and about life in general as well as having social problems, essentially means isolation and difficulties with reference to family and social relations^8^.

Due to the aforementioned, health and safety regulations, directives and procedures have been produced in Brazil to improve working conditions and the health of workers^9^ ^-^ ^11^.

Bad working conditions are detrimental for: processes aimed at improving working conditions, for those to whom care is being given, for health institutions and principally for health workers^12^. Are there connections between the effects related to nurse's work in clinical surgery departments at university hospitals and their physical, social and psychological suffering and pain?

The purpose of this paper was to explore the effects of work on the health of nurses working in clinical surgery departments at university hospitals and their connection with their physical, social and psychological suffering and pain. The theory is that the work carried out in clinical surgery departments produce negative effects which causes physical, social and psychological harm.

This study was necessary based on the then current state of play and through looking at the results, so that a discussions can be had with a view to improving medical working conditions which in turn will improve the lives of nurses and aid towards the construction of knowledge in this area.

## Method

This was a quantitative transversal study carried out in clinical surgery departments at four university hospitals located in the south of Brazil. They were identified with Arabic numerals. This region has a total of six university hospitals according to the Ministry of Education. Four of them are located in the state of Rio Grande do Sul where the research took place.

We observed 95 nurses working in clinical surgery departments who were distributed as follows: 11 at HU-1 that had 46 bed for inpatients in for surgery, 71 at HU-2 that had 221 clinical surgery department beds, seven at HU-3 that had 37 clinical surgery department beds and six at HU-4 that had 25 clinical surgery department beds. 

The research was authorized and given a favorable opinion by the Ethics Committee Protocol number CAAE 02505512.4.0000.5505. This was in accordance with Resolution 466/12 of the National Health Council.

The inclusion criteria was: being a nurse, doing either the morning (07-13h), afternoon (13-19h) or night (19h to 07:00) shifts and having work for at least one year as a nurse. We excluded: those that were on leave, hadn't been or were not working for what reason or who worked half shifts. 

The calculations of the sample were done through considering the level of significant at 0.05 with a statistic power of 95% and an alfa of 5%. The selection was random and the use of these parameters produced a sample size of 65 nurses.

Data collection occurred between July 2012 and January 2013 by four researchers who were qualified academics in the area of nursing. Data was both collected in person and done online in a trustworthy manner. The nurses received personal participation requests and were told about the objectives of the study and the voluntary nature of their input. Consent for participating was given through their signing Consent Forms, one copy of which was kept by the researcher and the other by the participant.

Developed by the authors of this papers and based on the Evaluation Scale of Relational Harm at Work (EADRT), a questionnaire was drawn up and given to the nurses (having closed questions) covering the following: health, work and general life habits, age, sex, time working at the clinical surgery department, shift work, option for shift work, training at work, other work, marital status, job satisfaction and satisfaction with salary, involvement in any accidents at work, leisure activities and the practice of any physical activities.

EADRT is a scale that has seven points and Likert is at the top: 0= not once, 1= once, 2= twice, 3= three times, 4= four times, 5= five times, 6= six times or more. The 29 items on the scale are grouped by factors: Physical Injuries (12 items), Psychological disorders (10 items) and Social harm (seven items). This is one of the four scales that make up the Work and Risk of Illness Itinerary (ITRA) which is a tool created in Brazil which assesses the connections between work and risk of illnesses. The third version of this tool was used in this study which is in the public domain^8^. We obtained permission from the creators to use it in our study.

The digitized data was typed up twice and statistically analyzed with Predictive Analytics Software, from SPSS Inc., Chicago - USA), version 15.0 for Windows.

Based on advice from the authors we interpreted the results through looking at the general averages and the percentage of respondents in the average intervals. The results were classified as: tolerable (positive evaluation - points below 1.99), critical (moderate evaluation - points between 2.0 and 3.0), serious (moderate evaluation for frequency - points between 3.1 and 4.0) and the presence of occupational illnesses (the most negative evaluation - points above 4.1). The proposed items covering situations related to health and their appearing and repeating themselves at moderate levels meaning the onset of an illness^8^. Based on the aforementioned the data was categorized as: non-illness (classified as tolerable) and illness (classified as critical/serious/presence of a disease).

The qualitative variables were described through absolute and relative frequency and were associated with the EADRT factors using the Chi-Sqaure and Fisher's exact tests. We carried out the Kolmogorov-Smirnov test to check the adherence of the data to the normal distribution when we analyzed the quantitative variables. The variables of age and time at work at the units were within what was considered normal and were described through averages and deviations from the norm. The correlation between the EADRT factors (physical, social and psychological suffering and pain) was analyzed through the Spearman's Correlation Coefficient model.

For all the tests 5% significant level was used (p<0,05) based on the reliability of EADRT and all of the scale factors were evaluated through Cronbach's alfa coefficient.

## Results

The following were the characteristics of those that took part in the study: 65 working nurses (in clinical surgery), mainly women (87.8%, n=57), average age 40.62 (± 8.82), average time in profession in clinical surgery 7.91 (±7.12), and who worked in shifts (morning - 32.3%, n=21, afternoon - 30.7%, n=20 and night - 34.9%, n=24). We noted that: the majority had partners (53.8%, n=35), were satisfied with their job (93.85%, n=61), opted for shifts that allowed them to do their activities (81.5%, n=53), had undergone training (64.62%, n=42) and had another job (76.92%, n=50). The majority of them: had been in a work accident (50.8%, n=35), carried out leisure activities one or more times per week (90.8%, n=59) and practiced physical activity (52.3%, n=34).

Looking at how work effected the health of the nurses we noted that physical injuries was the factor that showed the highest average (2.17±1.04), followed by social harm (1.52±1.24) and then psychological problems (1.18±1.15). The critical classification for physical injuries was predominant (43.1%, n=28), then tolerable for the social harm factor (64.6%, n=42) and finally psychological problems (83.1%, n=54). The EADRT had a consistency of internal satisfaction (Cronbach> 0.70), as shown in Table 1.

**Table 1:** Cronbach's alfa distribution and the classification of nurses packed in clinical surgery departments at university hospitals having physical injuries or suffering from social and psychological pain. Southern Region, Brazil, 2013 (N=65)

Factor	Classification				Cronbach's Alfa
	Tolerable	Critical	Serious	Disease	
Physical Injuries	24(36.9%)	28(43.1%)	10(15.4%)	3(4.6%)	0.82
Social Problems	42(64.6%)	16(24.6%)	4(6.2%)	3(4.6%)	0.85
Psychological Disorders	54(83.1%)	6(9.2%)	2(3.1%)	3(4.6%)	0.92

Amongst the ill nurses, 26 (57.8%) had problems that were connected to their work and 19 (42.2%) had more than one type of work related problem. 63.1% were physically ill, as shown in Table 2 and the physical problems that had the highest averages were pains in the legs (3.85±1.99) and back pains (3.82±2.29). Social Problems did not feature high on the list of factors that caused illnesses however the highest average under this category was wanting to be alone (2.18±2.14) and generally being impatient with people (1.85±1.81). Under the factor of Psychological Disorders, we noted the highest percentage for those that were not made ill because of it. Nevertheless the highest averages under this factor was not being in a good mood (1.85±1.82) and being irritated by everything (1.63±1.81).

**Table 2:** Distribution of the Physical, Social and Psychological problems for the clinical surgery department nurses showing the percentage of those that did and did not become ill due to the given factors. Southern Region, Brazil, 2013 (N=65)

Factor	Did not become Ill	Became Ill
Physical Injuries/harm	24(36.9%)	41(63.1%)
Social Problems	42(64.6%)	23(35.4%)
Psychological Disorders	54(83.1%)	11(16.9%)

We noted significant statistical differences between psychological problems and work accidents (p=0.018) and the option to work shifts (p=0.035). This was associated with the categories Ill and Not becoming Ill for the aforementioned three factors. We also noticed a connection between someone facing social problems and the practice of physical activity (p=0.036).

There were direct and moderate correlations between physical injuries and social problems (r=0.438, p<0.001) and between physical injuries and psychological problems (r=0.428, p<0.001). This was noted through the use of Spearmann's correlation model. There was a direct and very high correlation between social problems and psychological problems (r=0.804, p<0.001).

## Discussion

We noted that nurses working in clinical surgery departments in the south of Brazil, as a consequence of their work, became physically ill. The physical injuries category is the main indicator that can show that someone is suffering and this is an alert^8^. As a result of this, immediate short and medium term actions are needed which could help the nurses have a better quality of life, reduce the number of them being away from work and assist them in their work on a general basis.

In relation to the highest average found for the physical injuries category, this can be justified due to the characterization of the work process for clinical surgery department nurses. Being a nurse requires agility to carry out their work as well as the following attributes: they need to provide guidance to patients before and after operations, work with families, manage work shifts and work with the human resources department in relation to material and physical resources. In this context nurses have to produce their best which requires physical and mental health.

The organization of their work may explain their leg and back pains which causes them to be unwell. Studies done on nurses working in Intensive Care Units (UTI) in public and private hospitals and in the University of Turkey showed the same muscular and skeletal problems with pains in the legs and back. This suggested that working in UTIs is a main cause in comparison to other wards ^13^.

Lower back pains and varicose veins were mentioned as the main problems for nurses working in a public hospital in the north east of Brazil. These problems started when they started working on the hospital wards. This further shows that preventive actions are needed and nurses should fight for better working conditions^14^.

Someone's quality of life at work (p=0.010)^15^ ^)^ can be significantly improved where the person is not suffering from lower back pain. This was the conclusion of a piece of research done on nurses. The research also noted that muscular and skeletal problems reduces a person's ability to work^6^.

Muscular and skeletal problems increased from 28.35% in 2006 to 33.65% in 2010 due to poor posture and psychological problems. This was found in a study conducted on nurses between 2004 and 2010 in Taiwan^7^. In an American study with 361 nurses that provided critical care, 83% of them had a propensity to get muscular and skeletal lesions due to their work activity within one year. The nurses believe that the risk of getting these lesions was greater for their colleagues than for themselves^16^.

The nurses ought to look after themselves more and the area where they work. They often spend more time assisting their colleagues at their own detriment. This in turn causes problems for them^12^. A specific team should be put together to bring in programs designed to prevent ergonomic risks in hospitals^13^. There should also be massage treatment to alleviate lower back pain for nurses. These measures would go a long way towards improving nurses output ^17^.

Illnesses in this context could mean the following: problems in their daily lives and domestic routines, problems in relation to their leisure activities and an inability to work well. The consequences are: feelings of frustration and uselessness, coupled with pain, insomnia, mood swings, low self-esteem, depression, anxiety, not valuing oneself professionally and a propensity to suffer accidents and work^18^.

The theory that hospital work has an influence on nurses' health in clinical surgery departments was proven as we noted a connection between accidents at work and psychological problems. It is not possible to change the fundamental nature of nurse's work which is typically insalubrious. There are limits on how their work can be organized. However it is possible control the extent of the insalubrity as well as the dangerous aspects of the work and the shear exhaustion that can be caused. This can give back to nurses more energy and reduce their exposure to risk factors. Reducing the number of hours done on their shift would help in this process^5^.

Psychological fatigue based on problematic situations at their work had more impact on their quality of life compared with the actual nature of their work. This was the case even though this study showed that the majority of the nurses suffered from physical problems^19^.

It was difficult to assess emotional problems related to their work because these types of problems appear gradually^20^. One of the factors that can contribute to this is work shifts. The night shift causes physical difficulties when nurses need to do a day shift the following day ^3^.

This study found that individuals who did not practice physical activities were prone to isolation and had difficulties in relation to their family members and social problems. According to a Brazilian study involving 292,553 people aged 14 and over, one in five of them did not undertake any form of physical activity^21^. This study was similar to the aforementioned one. Not looking after oneself has a knock on effect in that the nurses' professional life may be harmed and they may also feel unmotivated both at work and in their homes. This can then lead to the onset of illnesses^22^.

Giving them the opportunity to choose their shifts will go a long way in providing professional work satisfaction. Our study showed that nurses who did not choose their shifts had negative feelings in relation to themselves and life in general (Psychological Disorders) which suggest a form of suffering on their part. We also noted strong correlations between presenting social problems and having psychological problems. This suggests that they are inter-related and they influence each other.

Becoming ill in a medical institution is due to many factors that need to be analyzed from the view point of the person who becomes ill. This requires a reductionist vision on the process of becoming ill. Bad working conditions and personal issues make nurses vulnerable to becoming ill. A wider understanding of this area is needed. This is necessary because there are multiple causes which are inter-related. This is also the case concerning the possibility of direct and indirect consequences for workers, their families and society as a whole^14^.

The results of this study are limited to its transversal scope. Reverse causality cannot be discarded. Uninvestigated themes such as the types of work accidents, whether sharp instruments were used and the type of body fluids that were involved, means that other lines of investigation can be opened.

## Conclusion

Based on our representative sample, we noted that physical illnesses were related to the work done by the majority of the nurses working in clinical surgery departments at university hospitals in the southern region of Brazil. The most amount of pain was registered in the legs and back. Physical Injuries/harm was classified as critical and the psychological and social problem factors were assessed as tolerable and correlated. Health problems for the nurses working in clinical surgery departments showed direct correlations, particularly between social problems and psychological problems which was very high.

We identified a significant connection between having social problems and the practice of physical activities. Psychological injuries had a significant connection with the variable accidents at work and the option to work shifts (respectively p=0.018 and p=0.035). The theory that work done by nurses in clinical surgery departments in medical institutions can be detrimental to their health was proven.

The results bring new scientific knowledge into this area meaning that planning should be undertaken to bring in preventive actions in order to improve nurses' health, particularly for those that work in the hospital system in the southern region of Brazil.
